# Exploring the long-term changes in the Madden Julian Oscillation using machine learning

**DOI:** 10.1038/s41598-020-75508-5

**Published:** 2020-10-29

**Authors:** Panini Dasgupta, Abirlal Metya, C. V. Naidu, Manmeet Singh, M. K. Roxy

**Affiliations:** 1 Centre for Climate Change Research, Indian Institute of Tropical Meteorology, MoES, Pune, 411008 India; 2 Department of Meteorology and Oceanography, College of Science and Technology, Andhra University, Visakhapatnam, 530003 Andhra Pradesh India; 3 Department of Atmospheric and Space Sciences, Savitribai Phule Pune University, Pune, 411007 Maharashtra India; 4 IDP in Climate Studies, Indian Institute of Technology Bombay, Mumbai, India

**Keywords:** Climate sciences, Ocean sciences, Planetary science

## Abstract

The Madden Julian Oscillation (MJO), the dominant subseasonal variability in the tropics, is widely represented using the Real-time Multivariate MJO (RMM) index. The index is limited to the satellite era (post-1974) as its calculation relies on satellite-based observations. Oliver and Thompson (J Clim 25:1996–2019, 2012) extended the RMM index for the twentieth century, employing a multilinear regression on the sea level pressure (SLP) from the NOAA twentieth century reanalysis. They obtained an 82.5% correspondence with the index in the satellite era. In this study, we show that the historical MJO index can be successfully reconstructed using machine learning techniques and improved upon. We obtain a significant improvement of up to 4%, using the support vector regressor (SVR) and convolutional neural network (CNN) methods on the same set of predictors used by Oliver and Thompson. Based on the improved RMM indices, we explore the long-term changes in the intensity, phase occurrences, and frequency of the winter MJO events during 1905–2015. We show an increasing trend in MJO intensity (22–27%) during this period. We also find a multidecadal change in MJO phase occurrence and periodicity corresponding to the Pacific Decadal Oscillation (PDO), while the role of anthropogenic warming cannot be ignored.

## Introduction

The Madden Julian Oscillation (MJO) is one of the most critical and complex weather phenomena in the tropics^1,2^. The MJO is manifested through the slow (5 ms^-1^) eastward propagating convective system, which usually originates over the western Indian Ocean and dies out over the cold sea surface temperature (SST) beyond the central Pacific. MJO is the source of the predictability in between the weather and climate time scales (i.e. beyond three weeks to six months) in the tropics. Although being a tropical phenomenon, MJO has a significant impact on global weather and climate. Among the diverse processes in the ocean–atmosphere system across the globe, MJO is exceptional due to its strong association with both weather and climate^3^.

For the real-time tracking of the eastward propagating MJO convective signals, Wheeler and Hendon^4^ derived the Real-Time Multivariate MJO Index (RMM) based on satellite-derived outgoing longwave radiation (OLR) and zonal winds at 850 and 200 hPa from NCEP reanalysis datasets (hereafter mentioned as WH04 RMM). The use of satellite OLR along with zonal winds is a crucial factor in the definition of the WH04 RMM index as it explains the amount of convective activity associated with the MJO circulation. This factor distinguishes the WH04 RMM index from other solely dynamical or convection based MJO indices like velocity potential index^5^ and bimodal ISO indices^6^, respectively. WH04 RMM index explains the strength and location of MJO signals in the tropical belt. WH04 RMM has been widely used for studying the MJO in the current satellite period (1979 onwards) and has been instrumental in the findings of many aspects of MJO variability. However, WH04 RMM index is not available before the satellite era, as satellite based OLR data facilitates its computation.

As the understanding of the mechanism and characteristics of MJO gradually developed over the last few decades, some questions arise. How does the interannual-to-decadal variations (low-frequency variability) of the underlying tropical SST modulate the MJO activity? How has the long-term inhomogeneous trends in the underlying tropical SSTs affected the MJO so far? What will be the MJO characteristics in the future warmer climate? To answer these questions, the scientific community needs reliable MJO data for an extended period and climate models which can simulate the MJO with fidelity.

Using the zonal winds (a dynamical proxy of MJO) from NCEP-NCAR reanalysis, Jones and Carvalho^7^ showed that there were significant trends in the intensity of MJO in boreal winter and summer, during the period 1958–2004. They also observed a significant trend in the number of boreal summer MJO events. Pohl and Matthews^8^ using the WH04 RMM index observed that the MJO lifetime in October-December and March–May are related to the underlying ENSO conditions. During the positive ENSO phase, MJO is generally faster and has a shorter lifetime, and during the negative ENSO phase, MJO is relatively slower and has a longer lifetime. Their study also used a dynamical proxy of MJO during 1950–2004 and showed that there was a significant shift in MJO amplitude around the year 1975 (at the same time as the shift in the ENSO time series). They showed that after 1975, the MJO amplitude is significantly higher than the earlier period and became independent of ENSO. To study the variability of the MJO on a wide range of time scales, Jones and Carvalho^9,10^ prepared an MJO index (similar to WH04) dataset for an extended period (1880–2008) using stochastic Markov model from observed SST anomalies. In that study, they observed a significant decadal shift in the number of MJO events per year. They found that the number of MJO events per year (1973–2008) were significantly more (3.9–4.6 events yr^-1^) than the earlier period (1948–1972) (2.6–3.4 events yr^-1^). This study was the first attempt to prepare an extended record of the MJO index. Using a relatively simplistic approach, Oliver and Thompson^11^ prepared a reliable 100 years data of the MJO index (WH04 RMM) from 20th Century Reanalysis V2 sea level pressure (SLP) datasets. Hereafter in the text, we denote the Oliver and Thompson^11^ study as OT12 and the derived MJO index as the OT12 index.

OT12 identified twelve locations in the tropical regions where SLP time series capture the MJO variability (WH04 RMM) reasonably well and are well supported by the long-term station observations. OT12 prepared the long-term MJO index data by employing multivariate linear regression (MLR) of WH04 RMM on 24 predictors (SLP data at 12 locations and their Hilbert transforms). They observed a weak, increasing trend in MJO amplitude over the past century (13% increase over the last century). However, Oliver^12^ observed that the realism of the reanalysis products, such as the twentieth century V2 reanalysis, might be impacted by the scarcity of observations or the changing number of observations that had been assimilated by the reanalysis system. Oliver^12^ noticed that the trend in MJO amplitude is dependent on the choice of input SLP locations and can vary from zero to 30% according to the choice. Therefore, the study argued that the trend in the MJO amplitude might have originated due to the errors in the reanalysis products.

Using the JRA55 reanalysis data, Klotzbach et al.^13^ derived the WH04 MJO index in their study where they noticed an emerging relationship between the MJO amplitude and QBO phases in the context of anthropogenic global warming^14^. The relationship between MJO and QBO has also been reported by other studies^15^. Klotzbach et al.^13^ observed a significant long-term increasing trend in MJO amplitude from the JRA55 reanalysis dataset.

As the span of the satellite-based database has grown, many new insights about MJO have been revealed. Recent studies have observed significant opposite trends in the number of active MJO days over the maritime continent (RMM phase 5, 6, and 7) and the Indian Ocean (phases 1, 2, and 3) in boreal winter during the satellite period from 1979–present^16–18^. It has been found that the lifespan of MJO is becoming longer and MJO is becoming slower over the maritime continent during this period. The opposite trends in lifespan and phase speed of MJO are observed over the Indian ocean. These studies argue that the variability in the number of active MJO days and propagation speed is related to the decadal variability and anthropogenic trends in the tropical Pacific.

The summary of the studies carried out using the observations, reanalyses, and the extensive database of the MJO prepared by statistical techniques are as follows: 1. There is a weak, increasing trend in the MJO amplitude during the past century. 2. The number of boreal summer MJO events is increasing during this period. 3. The number of MJO days and the propagation speed of MJO over the maritime continent and the Indian Ocean have an opposite trend during the recent period (1979–present).

The influence of the long-term tropical SST changes on MJO variability has been studied from the perspective of moisture mode theory framework with the help of state-of-the-art climate models^19–22^. Arnold et al.^23^ noticed that intraseasonal OLR variance increased with the increase in tropical SST from his aquaplanet experiment using the NCAR community Atmospheric model. Rushley et al.^24^ examined the projected changes of MJO in response to greenhouse-gas induced warming during the twenty-first century using five CMIP5 models. They observed that the MJO related precipitation variation is likely to be stronger in future warmer climates. MJO phase speed is likely to increase (1.8–4.5% K^–1^) with a decrease in zonal wavenumber (1.0–3.8% K^–1^). Maloney et al.^25^ synthesized the present understanding of the projected changes in the MJO under anthropogenic warming. This study also highlighted that the correctness of the projected changes in SST is crucial for the future projections of the MJO. Between the climate models with good MJO fidelity, there is disagreement on the MJO related wind variability in future climate^25^. Tropical SST has been warming throughout the twentieth century and is experiencing accelerated warming in recent decades. In the last two decades, many attempts have been made to track the changes in the MJO related to these SST warming in the last century. This knowledge is crucial for future MJO projections as well. However, the role of low frequency natural variability (e.g. PDO) on MJO is still not well understood.

In the new era of computing, machine learning is proving to be an excellent tool in handling complex non-linear problems in climate science. Machine learning algorithms are data-driven, which means they learn nonlinearities and complex relations in the large datasets and can be used further for optimization, classification, and prediction purposes. Support vector regression (SVR) is a popular machine learning algorithm with less overfitting problems. Very recently, deep learning emerged as a powerful tool in the field of machine learning and artificial intelligence^26^. Support vector machines and convolutional neural networks (CNN) both are excellent techniques for nonlinear regression and classification.

OT12 extended the WH04 MJO index for the pre-satellite period using a multilinear regression technique. The OT12 MJO index has been thoroughly validated and used by later studies^13,27,28^. However, we believe that there exists a scope for further improving the historical index of OT12 using nonlinear regression methods of machine learning. Also, the possibilities of historical reconstructions of climate data using machine learning approaches are yet to be explored. OT12 MJO index components (RMM1 and RMM2) match 82.4 and 82.5%, respectively, with the original RMM components in the known period (1979–2015). In this study, we aim to improve the historical index, using the MLR, SVR, 1dimensional (CNN-1D), and 2 dimensional (CNN-2D) techniques. Here, we have also explored how different methods of nonlinear regression perform for historical reconstruction of WH04 RMM. Unlike the OT12, we apply all the techniques on the ensemble average SLP time series. We have also used the WH04 RMM index derived from JRA55 reanalysis, which was used by Klotzbach et al.^13^. Using all these MJO indices derived from different regression techniques and reanalyses, we summarize the changes in MJO characteristics in the last century. We have focused on the following questions in our study. 1. Which of the applied nonlinear methods are suitable for historical reconstruction of WH04 RMM ? 2. What are the changes in the amplitude, phase occurrences, and the frequency of MJO events in the last century (1905–2015) ? We confine our study with boreal winter MJOs because the mechanism involved for boreal summer MJOs is different and can be investigated in a separate study. 3. What is the influence of the low-frequency multidecadal SST variability (PDO) on MJO ?

## Results

### Improvement in the correlation of derived MJO indices with original WH04 MJO indices

Our newly derived RMM1 and RMM2 are standardized based on the 1979–2008 period (satellite era) (Supplementary Figure 1). We calculated the correlations between original WH04 RMM and the RMM from OT12, MLR, SVR, CNN-1D, and JRA55 for the training (1979–2008) and two test periods (1974–1978, 2009–2015) in Table 1. We do not explicitly mention the correlation in the training period (1979–2008), but we mention the correlation for the period 1979–2015 when there is no discontinuity in the WH04 MJO index. Within the period 1979–2015, the major part comes under the training period (1979–2008), so these correlations are representative of training correlations of the models. The MJO index derived from our MLR is very similar to the OT12 index which is computed based on 54 ensembles of 20CRV3 reanalyses. The respective correlation coefficients between (WH04-RMM1, WH04-RMM2) and (MLR-RMM1, MLR-RMM2) are (0.83, 0.83) for the training period (1979–2015) and are (0.83, 0.81) for the test1 period (1974–1978). However, we notice that for the test2 period, the correlation coefficients are relatively lower (0.79, 0.80). Similarly, for (OT12-RMM1, OT12-RMM2) the training, test1 and test2 correlations are (0.82, 0.82), (0.83,0.83), and (0.77, 0.76).

**Table 1:** Correlation (*r*) between the original WH04 RMM and predicted RMM for the total period (1979–2015) and two test periods (1974–1978, 2009–2015).

Model	RMM1			RMM2		
	Total period (1979–2015)	Test1 (1974–1978)	Test2 (2009–2015)	Total period (1979–2015)	Test1 (1974–1978)	Test2 (2009–2015)
OT12	0.824	0.831	0.775	0.825	0.829	0.764
MLR	0.829	0.833	0.79	0.831	0.809	0.798
SVR	0.839	0.837	0.797	0.84	0.813	0.804
CNN-1D	0.864	0.861	0.824	0.871	0.826	0.823
CNN-2D	0.931	0.87	0.873	0.922	0.844	0.841

We employed an SVR model with the same input as MLR. We obtained (0.84, 0.84) correlations in the training period. For the test1 period, correlation values are (0.83, 0.81) and for the test2 period, correlation values are (0.80, 0.80). We observe a slight correlation improvement in the SVR-RMM model relative to MLR-RMM and OT12 RMM. The improvement in correlation is statistically significant at 0.05 significance level according to Zou’s correlation comparison test^29^.

Meanwhile, we observe a significant improvement in the test correlation using the CNN-1D model. Our CNN-1D model is designed to predict the MJO index from the SLP time series at 12 locations, and their Hilbert transforms for the last 120 days. We obtained correlation values (0.86, 0.87) in the training period (Table 1). For the test1 period, correlations are (0.86, 0.826) and for the test2 period, the correlations are (0.82, 0.82). We, therefore, observed an improvement up to 0.04 correlation in the training period and up to 0.05 correlations in the test1 and test2 period for both RMM1 and RMM2. The improvement in correlation is statistically significant at 0.05 significance level according to Zou’s correlation comparison test^29^. We assume that this improvement can be useful for preparing a more reliable historical reconstruction of the RMM index.

We applied CNN-2D technique on the SLP maps in the tropics. We obtained the most improved correlation in the training (0.93, 0.92), test1 (0.87, 0.84) and test2 (0.87, 0.84) periods using this technique (Table 1). However the noise in the SLP data, originating from a lack of observations in the historical period, reduces the reliability of the SLP in the past^12^. This noise can potentially contaminate the historical reconstruction of the RMM indices. Therefore, those grid points in the reanalysis where the density of observations is large should be considered for the historical reconstruction. Historical reconstruction using fewer predictors are hence favorable for this reason. Hence, we omit this technique for a full historical reconstruction of WH04 RMM.

### Lag correlation between RMM1 and RMM2

**Figure 1:**
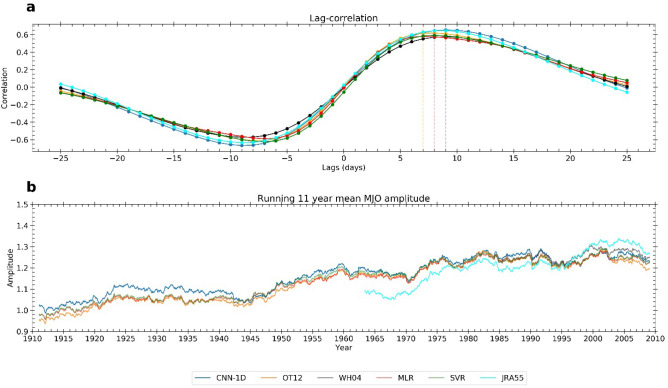
(**a**) Lead-lag correlation between RMM1 and RMM2 for CNN-1D (blue), OT12 (orange), WH04 (black), MLR (red), SVR (green) and JRA55 (cyan) index. For MLR, SVR, and OT12 index, maximum correlation is observed at a lag of 7–8 days. For CNN-1D, JRA55, and WH04 maximum correlation is observed at a lag of 9 days. (**b**) Running 11-year mean MJO amplitude for CNN-1D, OT12, WH04, MLR, SVR, JRA55 MJO index (blue, orange, black, red, green and cyan respectively). Long-term trend values according to the different MJO indices range between 0.23 and 0.27 per century, based on 1905–2015 data. The trend indicates a 22–27% increase of MJO amplitude per century.

As depicted by Wheeler and Hendon^4^, WH04 RMM2 lags WH04 RMM1 with a maximum correlation of 0.56 at a lag of 9 days. For the newly derived RMM1 and RMM2 (Table 2) indices, we observe that MLR-RMM2, OT12-RMM2, and SVR-RMM2 have a maximum correlation with respective RMM1s at a lag of 7–8 days (Fig. 1a). However, WH04-RMM2, CNN-1D-RMM2, and JRA55-RMM2 have a maximum correlation at a lag of 9 days with the corresponding RMM1s (Table 2). Accurate lag correlation between RMM1 and RMM2 is crucial for determining the correct MJO phase locations. We compared the phase agreement between the newly derived MJO index and the WH04 RMM in the period 1979–2015. We observe a 1–6% increase in the phase agreement in CNN-1D index with respect to the OT12 index (Supplementary Fig. 2).

**Table 2:** Lead-lag correlation between RMM2 and RMM1 for different MJO indices.

MJO indices	RMM2 lags RMM1 (days)	Max correlation values
WH04	9	0.57
OT12	8	0.61
MLR	7	0.58
SVR	8	0.59
CNN-1D	9	0.65
JRA	9	0.64

### MJO propagation during the satellite and pre-satellite era

Following OT12, here we verify the MJO propagation for the newly derived RMM index (CNN-1D-RMM1 and CNN-1D-RMM2) for the satellite (1979–2008) and pre-satellite period (1952–1978) (Supplementary Fig. 3). We prepared composites of cloud fraction over the global ocean for the active MJO days with amplitude more than 1.5 times of its standard deviations, based on 1979–2008 period (the value approximately is equal to 1.0 for WH04-RMM). We observe a clear MJO propagation in the cloud fraction composites of CNN-1D RMMs. We find that the cloud fraction in phases 7 and 8 during the pre-satellite (1952–1978) period are comparatively less than those during the satellite era (1979–2014) (Supplementary Fig. 3). This indicates that the MJO propagation from the western to central Pacific were weak during this period. We notice the same for MLR, SVR, JRA55 and OT12 RMM (not shown).

### Changes in boreal winter MJO amplitude, phase occurrences and number of MJO events over the twentieth century

Based on the derived MJO indices from different techniques (MLR, SVR, CNN-1D), OT12 index and JRA55 RMM index, we have tried to synthesize the observed changes in boreal winter MJO during the twentieth century (1905–2015). In the following section, we describe the changes in MJO amplitude, phase occurrences and the number of MJO events.

### MJO amplitude

Earlier studies noticed a weak trend in the MJO amplitude during the twentieth century^7,9,11^. However, Oliver^12^ points out that the trend in the MJO amplitude might be generated from the scarcity and the changing number of observations assimilated in the reanalysis system. Hence, the reanalysis data must be interpreted carefully and we should consider the fact that there is an uncertainty in the long-term trends of the MJO amplitude. Regardless, we have summarized the trend in the MJO amplitude based on the databases of different MJO indices prepared from different methods and reanalyses.

Time series of the MJO amplitude (11-year running mean) based on the CNN-1D, OT12, WH04, MLR, SVR, and JRA55 MJO indices are plotted in Fig. 1b. The long-term trend in MJO amplitudes are 0.23 (CNN-1D), 0.27 (OT12), 0.27 (MLR), 0.27 (SVR) per century during the entire period 1905–2015. This corresponds to a 22–27% increase of MJO amplitude per century. We notice that the trend values in CNN-1D, OT12, MLR, SVR and JRA55 MJO amplitudes vary from 0.04–0.43 (3–40%) per century during 1959–2015. The trend is maximum in the JRA55 MJO index (40%) and minimum in the OT12 MJO index (3%) during 1959–2015 period.

**Figure 2:**
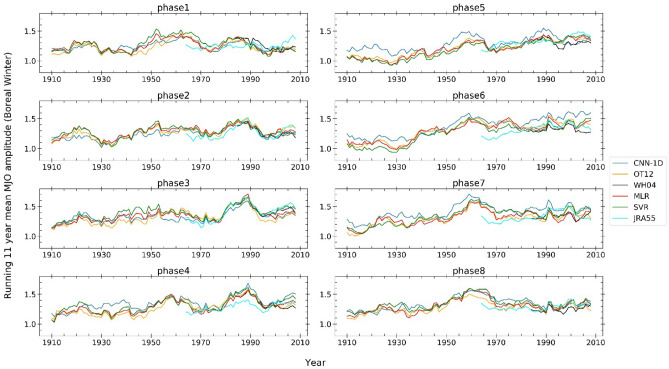
Running 11-year mean boreal winter MJO amplitude at the eight MJO phase locations, for CNN-1D, OT12, WH04, MLR, SVR, JRA55 MJO index. The long-term linear trends in boreal winter MJO amplitude are maximum at MJO phase locations 5 (Maritime Continent, 0.32 per century) and 6 (West Pacific, 0.35 per century).

We further explore the trends in the MJO amplitude (11-year running mean) during the boreal winter (Supplementary Fig. 3). We explore the trends separately at eight MJO phase regions during boreal winter in Fig. 2. We calculated the seasonal average MJO amplitude at each of the MJO phases and computed the 11-year running mean time series. We find that the trends in the MJO amplitudes at different RMM phase locations are not similar during boreal winter. The most significant and large trends in the MJO amplitude are present at phase 5, 6, and 7 regions. The long-term trend in the mean CNN-1D, OT12, WH04, MLR, SVR and JRA55 MJO amplitude at RMM phase locations 3, 4, 5, 6, and 7 during boreal winter are 0.22, 0.22, 0.32, 0.35 and 0.22 per century. The trends are maximum at RMM phase locations 5 and 6.

### MJO phase occurrences

**Figure 3:**
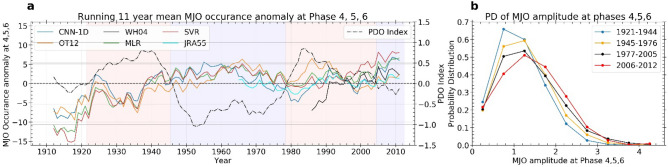
(**a**) Running 11-year mean boreal winter MJO occurrence anomaly at phase 4, 5, and 6 regions and the PDO index. MJO occurrence anomaly at phase locations 4, 5, and 6 according to CNN-1D, OT12, WH04, MLR, SVR, JRA55 MJO index (blue, orange, black, red, green and cyan respectively). Black line represents the running 11-year mean PDO index. (**b**) probability distribution (PD in %) of boreal winter MJO amplitude at RMM phase locations 4, 5, and 6 (over the Maritime Continent and west Pacific) for warm phases (1921–1944 and 1977–2005) and cold phases of PDO (1945–1977 and 2006–2012).

Another measure of MJO activity is the number of phase occurrences or active MJO days at different MJO phase locations as used by Yoo et al.^16^, Lin et al.^30^. Recently, Yoo et al.^16^, Junyi et al.^17^ and Roxy et al.^18^ reported that there are significant trends in the occurrences of the MJO phases in boreal winter (November–April) during 1981–2018. Here, the occurrences of MJO phases are calculated by counting the number of days when MJO amplitude is above a certain threshold value at a given phase location during the boreal winter season. We select the threshold as 1.5 times the standard deviation (1979–2008) of the MJO index (which is roughly 1.0 for WH04 MJO index). The 11-year running mean of MJO occurrences at phase 4, 5, 6 is shown in Fig. 3a. The time series of MJO occurrences at phase 4, 5, 6 for different MJO indices are subtracted from their long-term mean phase occurrence values. We notice an out of phase relationship between the MJO phase 4, 5, 6 occurrences and winter mean (November–April) PDO index. MJO occurrences at phase 4, 5, 6 are above normal during the negative phases of PDO and below normal during the positive phase of PDO. Correlation values between detrended MJO occurrences at phase 4, 5, 6 and detrended winter mean PDO index (also their 11-year running mean time series) are presented in Table 3.

**Table 3:** Pearson (*r*) and Spearman’s rank correlation (*ρ*) between MJO phase 4, 5, 6 occurrences (according to CNN-1D, OT12, WH04, MLR, SVR, JRA55 MJO index) and PDO index along with the (*p *value). The correlation coefficients for WH04 and JRA55 indices are obtained from the available data period.

Models	CNN-1D		OT12		WH04		MLR		SVR		JRA55	
	*r*	*ρ*	*r*	*ρ*	*r*	*ρ*	*r*	*ρ*	*r*	*ρ*	*r*	*ρ*
Winter mean PDO index & MJO 4, 5, 6 phase occurrences	− 0.13 (0.17)	− 0.13 (0.17)	− 0.18 (0.05)	− 0.20 (0.04)	− 0.43 (0.00)	− 0.36 (0.02)	− 0.22 (0.01)	− 0.23 (0.01)	− 0.19 (0.05)	− 0.18 (0.06)	− 0.12 (0.36)	− 0.10 (0.42)
11 year running-mean PDO index & Phase 4, 5 ,6 occurrences	0.40 (0.00)	− 0.38 (0.00)	− 0.28 (0.00)	− 0.25 (0.01)	− 0.22 (0.28)	− 0.21 (0.31)	− 0.37 (0.00)	− 0.37 (0.00)	− 0.34 (0.00)	− 0.36 (0.00)	− 0.28 (0.05)	− 0.26 (0.06)

We find that the occurrences of the MJO phases 4, 5, and 6 during boreal winter is weakly correlated to the PDO index. The correlation values range between 0.13–0.23 for different indices except for JRA55 and WH04, which are not available for the entire period. The correlation becomes significant when we consider only the low-frequency part of the signal (11-year running mean signal). In that case, the correlation coefficients range between 0.3–0.4 for the different indices with *p *value < 0.01. This means that the occurrences of the MJO phases 4, 5, and 6 have a statistically significant multi-decadal mean shift following the PDO phases (Fig. 3a). Notably, in the timeseries, the 11-year running-mean of MJO occurrences at phases 4, 5, and 6 explain around 23% of the total variability of MJO occurrences at these phases. We also notice that there is a long-term trend in the MJO occurrences at phase 4, 5, 6. The average long-term trend from CNN-1D, OT12, WH04, MLR, SVR and JRA55 phase 4, 5, 6 occurrences is 10 days per hundred years (*p *value = 0.002). This trend majorly exists due to the low MJO occurrences at phase 4, 5, 6 during 1905–1945.

We identify the warm and cool phases of PDO from 11-year running mean winter PDO index based on Fig. 3a. There were two warm phases (1921–1944 and 1977–2005) and two cool phases (1945–1976 and 2006–2012) of PDO. For the four phases of PDO, we calculate the probability density (PD) of MJO amplitude at phases 4, 5, and 6 (Fig. 3b). We obtain the average probability density based on CNN-1D, OT12, WH04, MLR, SVR, and JRA55 MJO amplitude. We find a gradual flattening of the PDF of MJO amplitude over phase 4, 5, 6 which means the number of large amplitude MJO days are increasing and resulting in the amplitude trend at phase 4, 5, and 6 (Fig. 3b).

### Number of MJO events

**Figure 4:**
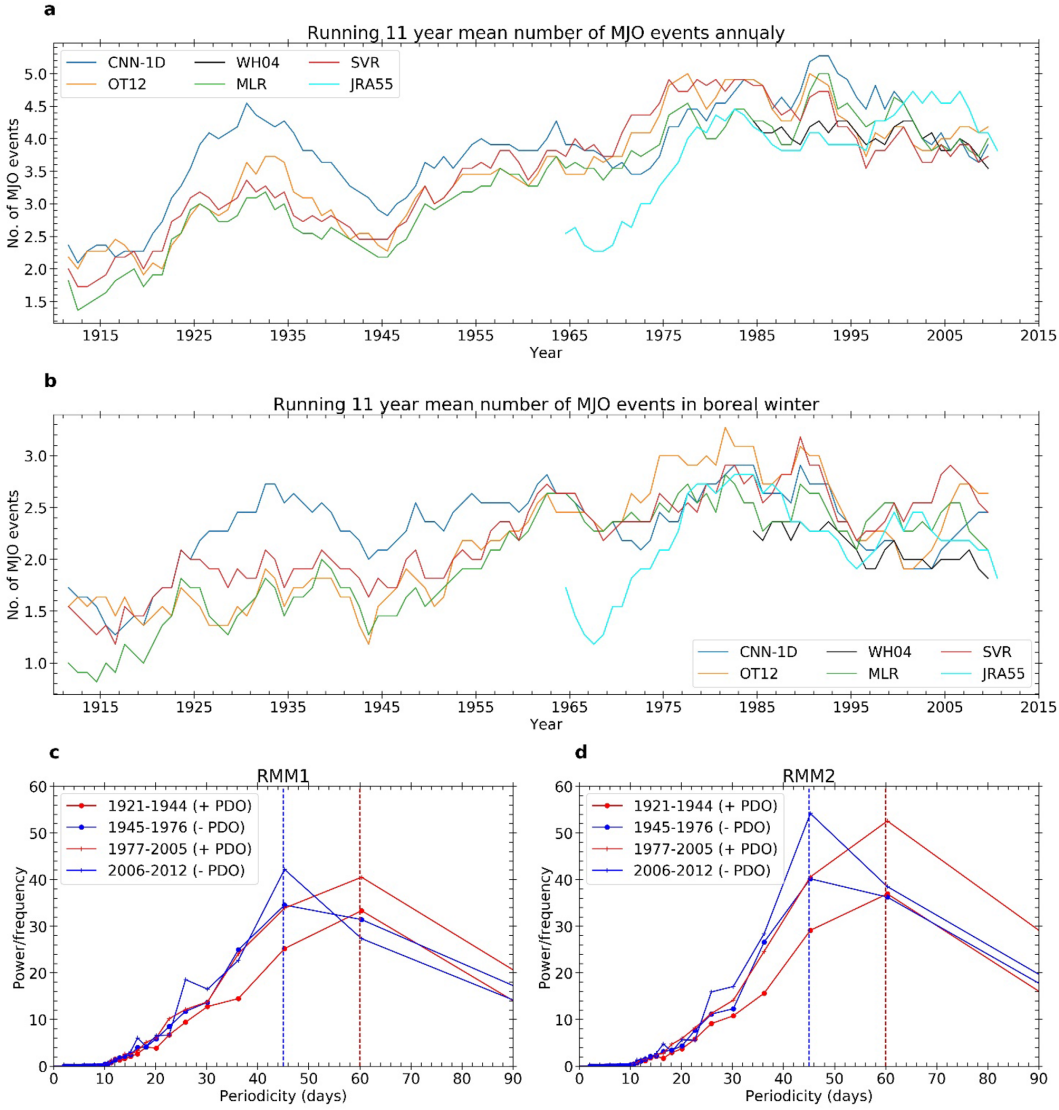
Running 11-year mean number of MJO events in (**a**) annual (upper panel) and (**b**) boreal winter (lower panel). Trend values are 2.32 (annual) and 1.26 (boreal winter) events per century, respectively. Average power spectrum of (**c**) RMM1 and (**d**) RMM2 during 1924–1944 (+ ve PDO), 1945–1977 (-ve PDO), 1977–2005 (+ ve PDO) and 2006–2012 (-ve PDO) boreal winter. A multi-decadal shift of the maximum power is observed between 45 and 60 days periodicity.

We have identified the MJO events based on the following criteria. During the events, the MJO amplitude should be larger than its standard deviation based on the 1979–2008 period at least for consecutive 15 days. Following the segment identification, the successive MJO cycles are separated. We consider those MJOs that at least cover 6 different phases after originating in the Indian Ocean (phase 1,2 and 3). If two MJO segments are separated by 5 or less number of days, we consider them as a single segment and analyse for the possibility of successive MJO events. Following this method, we identified the number of MJO events using CNN-1D, OT12, WH04, MLR, SVR, and JRA55 MJO index. We find a significant long-term trend (2.22 events per century) in the mean (based on multiple indices) number of MJO events per year, similar to ^7,10^ (Fig. 4a). The trend in the number of boreal winter MJO events is 1.13 events per century (Fig. 4b).

### Decadal power shift between longer and shorter timescales

The time scale of the MJO is 20–100 days. However, most of the energy in the MJO spectrum during the boreal winter is concentrated in the 30–60 days band. The RMM1 and RMM2 time series represent the propagating MJO signal. The two-time series have maximum coherence at the MJO time scale. The RMM1 pattern of MJO is related to the large-scale convection or suppression over the maritime continent, whereas the RMM2 pattern is related to the large-scale convection over the Indian Ocean and suppression over the western Pacific and vice versa. The periodicities of RMM1 and RMM2 denote the timescale of the repetition of MJO convective activity over the respective regions. Overall, the periodicity of MJO is dependent on both the lifespan and phase speed of MJO. To examine the multi-decadal change in MJO lifespan and phase speed, we have done the power spectrum analysis of RMM1 and RMM2 during the positive (1921–1944 and 1977–2005), and negative phases (1945–1976 and 2006–2012) of PDO based on our derived indices in Fig. 4c, d. We find a power shift in the MJO spectrum (power spectrum of RMM1 and RMM2) at a multi-decadal time scale. The peak frequency of MJO (the periodicity having the maximum power in the MJO power spectrum) is 45 days during the negative PDO phase and is 60 days during the positive PDO phase. The multi-decadal shift in the peak frequency suggests a change in the multi-decadal characteristics of the MJO lifespan and phase speed.

## Discussion

Three primary objectives of the present study are as follows. First, we explore the possibility of historical reconstruction of the MJO using machine learning algorithms, thereby improving the existing historical MJO index (OT12). Second, we aim to synthesize the long-term trends in MJO amplitude, phase occurrences, and the number of events based on the derived and existing historical MJO indices. Third, we explore the relationship between the low-frequency SST variability in the tropics (PDO) and the MJO.

Using the SVR and CNN-1D algorithm, we obtain a significant improvement in the known period correlation by 1% and 4%, respectively, compared to the existing OT12 MJO index. The model improvement is quantified based on 37 years of daily data (14,000 data points), where 1–4% correlation improvement means it performs relatively well and has less error than earlier. Regardless of the improvement in the reconstruction, our results highlight that machine learning methods can be successfully used for such historical reconstructions.

The historical MJO index reconstructed here using the CNN-1D method agrees better (especially at individual MJO phases) with the original WH04 index in the known period 1979–2014, compared to the earlier OT12 index. The OT12 index does loosely carry the relevant information about the link between MJO occurrences and PDO. However, we find that the PDO connection with the MJO phase occurrences (in the case of 11-year running mean time-series) is more evident in the CNN-1D index (-0.40) than the OT12 index (-0.28). That is, the CNN-1D index shows more robust interplay with the PDO than the OT12 index.

Based on our derived MJO index, OT12, and JRA55 MJO index, we find that the trends in MJO amplitude during boreal winter are not uniform at all the RMM phase locations. The trends in boreal winter MJO amplitude are maximum at RMM phase locations 5, 6, and 7, e.g., over the maritime continent and west Pacific. We also find that the occurrences of MJO activity at RMM phase locations 4, 5, and 6 during boreal winter are significantly related to the phases of the Pacific Decadal Oscillation (PDO). The MJO occurrences are relatively more during negative phases of PDO and less during the positive phases of PDO. The recent trend (1979–present) in the MJO occurrences at phase location 5, 6 and 7 is observed to be largely attributed to multidecadal variability of MJO. However, we cannot ignore the simultaneous role of anthropogenic global SST warming behind the trend in MJO occurrences and the PDO. We also find a significant multidecadal shift of the peak frequency (periodicity) of the MJO. This shift suggests that there is a significant change in the multidecadal characteristics of MJO lifespan and phase speed.

## Methods

### Predictors

In this study, we employ different statistical regression methods to reconstruct the MJO index for the twentieth century. We use the multilinear regression (MLR), support vector regressor (SVR), 1D, and 2D Convolutional Neural Network (CNN-deep learning) techniques. Oliver and Thompson^11^ calculated the MJO index using MLR although they calculated for each of the 54 ensemble members of 20CR V2 reanalysis SLP and prepared an ensemble mean OT12 index. Instead of deriving an index for each of the ensemble members, we derive the MJO index from the ensemble mean SLP data of 20CRV3 reanalysis. To compare our results with Oliver and Thompson^11^, we construct an MLR model to predict the MJO index from the ensemble mean SLP of 20CRV3.

For our MLR, SVR, and CNN-1D models the same set of SLP predictors as used by OT12 are employed. OT12 identified twelve locations across the tropical belt where the SLP time-series represents the MJO variability reasonably well (captures more than 80%). The locations of these predictors are listed in Supplementary Table S1. Following the OT12 methodology, we pre-processed the SLP data before performing regression. First, we removed the mean and first three harmonics from the SLP data to obtain the daily SLP anomalies. Then we applied a low-pass filter on the SLP anomaly with ten days cut-off frequency to remove the high-frequency waves (Kelvin and Rossby waves). Following that, we removed the previous 120-days mean from each timestep to remove the ENSO related variability. These preprocessed SLP data and the corresponding Hilbert transforms (total 24 predictors) are finally used as the predictors for the reconstruction of the MJO index using MLR, SVR, and CNN-1D models. However, we used the preprocessed SLP map between 30°N-30°S as the input to the CNN2D model. We have designed SVR and MLR to predict RMM1 and RMM2 of a particular day from that day’s predictor values. However, the CNN-1D is designed to predict RMM1 and RMM2 from the previous 120 days’ SLP values. The 120-days window is selected based on the timescale of the MJO process and also the way the WH04 RMM index is defined. The CNN-2D model is designed to predict RMM1 and RMM2 from that day’s SLP map. The SLP data is normalized using the min–max scaling method for the entire period (1905–2015) before performing SVR, CNN-1D, and CNN-2D regression.

### Regressors

We separated the known period of WH04 RMM, 1974–2015 into three subsets, 1974–1978, 1979–2008 and 2009–2015. We took 1979–2008 as the training period (same period as used by OT12) for our machine learning models, and the other two periods are considered as the test periods. We chose two test periods in two different times to ensure that the model performance is time-independent. We prepared our models using the training dataset and validated those models using the test dataset. Let us assume that the training dataset is $\left( {x_{1} ,y_{1} } \right),\left( {x_{2} ,y_{2} } \right),\left( {x_{3} ,y_{3} } \right),\left( {x_{4} ,y_{4} } \right), \ldots .,\left( {x_{i} ,y_{i} } \right),$ where $x$ represents the predictors (SLP) and $y$ represents the observed or target values (WH04 RMM index). Our main objective is to find a $f\left( x \right)$ (construct a model) from the training dataset which best predicts the test data. To find the $f\left( x \right){ }$, we used MLR, SVR, CNN-1D, and CNN-2D machine learning algorithms. In the following sections, we will discuss the details of these algorithms.

### MLR

Our MLR model is similar to the model used by Oliver and Thompson^11^. The model is described by Eq. 1.

1 $$y_{RMM} = \beta x\left( t \right) + \varepsilon$$

where $y_{RMM}$ is the predicted bivariate index at timestep $t$ derived from $x\left( t \right)$ (24 SLP predictors at that timestep). $\beta$ and $\varepsilon$ are regression coefficients and the error term respectively computed using the ordinary least square (OLS) approach.

### SVR

Support Vector Regression (SVR) is a popular machine learning algorithm for regression which reduces the problem of overfitting in OLS^31^. An example of the use of SVR in climate science is the long-lead prediction of ENSO Modoki index by^32^. In the OLS method, the main objective is to minimize the error between the model and observed values. Therefore, the objective function or loss function in the OLS method is,

2 $$Minimize : \sum \mu^{2} = \sum \left( {y_{i} - \beta x_{i} - \varepsilon } \right)^{2}$$

where $\mu$ is the error between the model and observed values. The model obtained from OLS is sensitive to the predictors (inputs or features). Errors in the measurement or non-stationarity of the predictors are amplified by the large coefficient values in the model and produce erroneous output. This problem is known as overfitting. An overfitted model predicts the training set well but fails to predict the test set. This problem of overfitting can be tackled by minimizing the absolute value or norm of coefficients ($\beta$) in the model and thereby making the model less sensitive to its inputs (flatness). The SVR algorithm follows this idea of minimizing the coefficient (the *l*2-norm of the coefficient) instead of minimizing the squared error. SVR ultimately finds a regression function $f\left( x \right) = \left\langle {w,x} \right\rangle + b$ , which best describes the observed output $y$ with an error tolerance $\varepsilon$ . $w$ and $b$ are the weighting vector and bias. The SVR algorithm restricts the absolute error of a model within a specified margin, called the maximum error or error tolerance ($\varepsilon$). $\varepsilon$ can be tuned to gain the desired accuracy of a model. The objective function of SVR is,

3 $$Minimize : \frac{1}{2} \left| {\left| w \right|} \right|^{2} + C \mathop \sum \limits_{i = 1}^{L} \left( {\xi_{i} + \xi_{i}^{*} } \right)$$

$$\begin{array}{l} Subject\;to\,: y_{i } - \left\langle {w,x_{i} } \right\rangle - b \le \varepsilon + \xi_{i} , \\ \left\langle {w,x_{i} } \right\rangle + b - y_{i} \le \varepsilon + \xi_{i}^{*} , \\ \xi_{i} \xi_{i}^{* } \ge 0 \\ \end{array}$$

ξ is the slack variable that measures the distance between the training samples and the margin $\varepsilon$ (amount of error outside $\varepsilon$ are allowed). Slack variables are those which turn an inequality equation into equality. The constant $C > 0$ determines the balance between the flatness of $f$ and the amount of error outside the margin $\varepsilon$ are allowed. The problem of optimization is solved using the Lagrange multipliers.

4 $$f\left( x \right) = \mathop \sum \limits_{i = 1}^{L} \left( {\alpha_{i } - \alpha_{i}^{*} } \right) k\left( {x_{i} ,x} \right) + b$$

where $\alpha_{i }$ and $\alpha_{i }^{*}$ are Lagrange multipliers and k is the kernel function which represents the nonlinear relationship between support vectors $(x_{i} )$ and input variable $\left( x \right)$. In the present study, the Radial Basis Function (RBF) kernel is used and the hyperparameters $C$ and $\varepsilon$ are taken as 0.1.

### CNN

The CNN algorithm is developed to recognize simple patterns within image datasets and thereby used for image recognition problems. CNN is a particular type of Artificial Neural Network (ANN) (also called Simple Neural Network) that relies on the linear operation called convolution^33,34^. For the ANN algorithm, neurons act as the operators (functions) having particular weights and biases (w and b). A neuron computes the dot product between its weights and its inputs $\left( {w.\,x} \right)$ and adds the product to its bias $\left( {w.\,x + b} \right)$. Then a nonlinear activation function is applied on the value (e.g., sigmoid, tanh, ReLU—Rectified Linear Unit), which gives the final output from a neuron. The primary goal of the ANN is to set these weights and biases to optimality, where the error (mean squared error or mean absolute error as the cost function) of the model is minimum. The optimization and backpropagation processes in ANN do the job of adjusting the weights and biases of the neurons.

The CNN is developed from ANN to work with two-dimensional input images. For a two dimensional CNN, weight is a 2D array (smaller in size than the input image) like its input, which is called a kernel or filter^26^. CNN can also work on one dimensional (1D), and three dimensional (3D) images where filters required are 1D and 3D, respectively. Through the convolution process, the dot product between the filter-sized patch of the input and filter is calculated, which is then summed up, producing a single value. The systematic application of the filter across an image produces a new image called the feature map. The feature map is then passed through the activation function and thereafter to the next convolution layer. If a filter is designed to detect any particular feature, then the systematic application of the filter across an image helps to detect the feature in that image. The basic idea of CNN is identifying the correct weights in the kernel matrix representing a critical feature associated with a particular output. Similar to ANN, the CNN model is updated through optimization and backpropagation algorithms (e.g. Stochastic Gradient Descent -SGD, Adam)^34,35^. The number of times the weights and biases are modified in a CNN model are called epochs, and the number of samples passed before an update of weights and biases are called the batch size.

1D and 2D CNN work on the same principle. The main difference is the size of the input data and how the feature detector (filter) slides over the data. In the following, we will discuss the architecture of 1D and 2D CNN used in our study.

### Architecture of CNN-1D model

**Figure 5:**
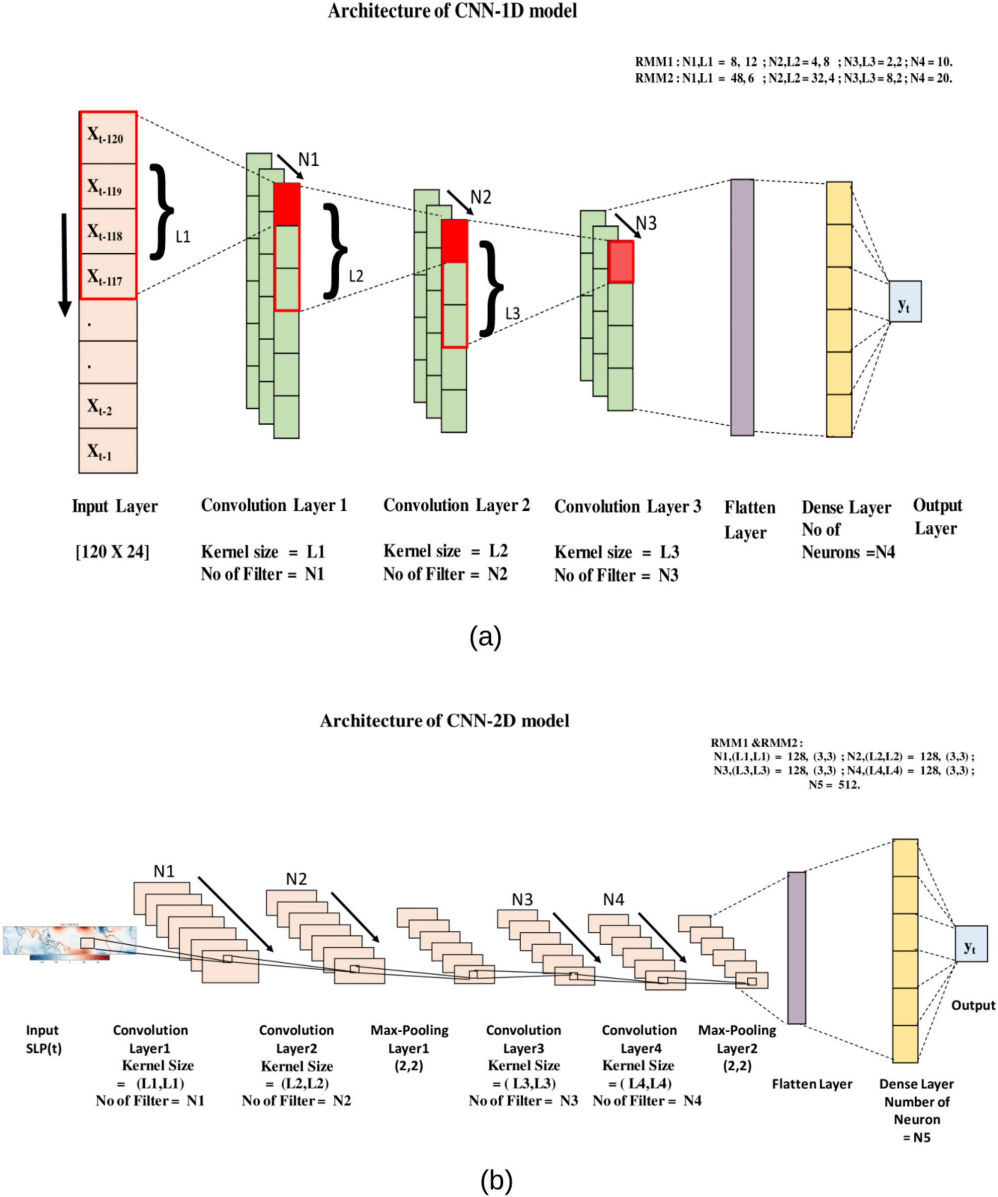
Architecture of CNN models used in the study. (**a**) CNN-1D model. (**b**) CNN -2D model*.*

A schematic for CNN-1D model architecture is described in Fig. 5a. The CNN-1D model has three convolution layers. The third convolution layer is linked to a fully connected dense layer, which is linked to the final output (a single value of RMM1 or RMM2). The first convolution layer consists of an N1 number of feature maps generated from the N1 number of 1D filters with the kernel size L1. For the second and third convolution layers, the number of the feature maps and kernel size are N2, L2, and N3, L3, respectively. We used the 'ReLU' activation function in our study. The CNN model is formulated for RMM1 and RMM2 separately. The number of convolution layers (in the present case 3), filters in each convolution layer (N), neurons in the dense layer, and the size of the kernels (L) are chosen based on the model performances in RMM1 and RMM2 prediction. We observed that three convolution layers are suitable for RMM1 and RMM2 regression. For RMM1 and RMM2, the values of N1, L1, N2, L2, N3, L3, N4 are mentioned in Fig. 5a. We used mean absolute error (MAE) as the loss function. We have used 'Adam' optimizer^35^ for the training of our CNN-1D model. We fixed the learning rate as 0.005, with the decay rate of learning as 1e-6. CNN-1D model is trained in 200 epochs with a batch size of 100.

### Architecture of CNN-2D model

In Fig. 5b, the schematic of CNN-2D is represented. The input of the CNN-2D model is an image of a tropical SLP anomaly at any particular time step, and the filters are 2D arrays. Our CNN-2D model has four convolution layers, two max-pooling layers with (2, 2) dimension, and a dense layer (Fig. 5b). The dense layer is connected to the final output (a single value of RMM1 or RMM2). The number of the filters and kernel size in each layer are mentioned in Fig. 5b. Similarly, as the CNN-1D model, we used mean absolute error (MAE) as the loss function. We employed the 'ReLU' activation function and the 'Adam' optimizer with a learning rate of 0.0001 having a decay rate of 1e-6. CNN-2D model is trained in 60 epochs with a batch size of 32.

### Validation

We prepared composites of cloud fraction for each of the MJO phases for the verification of our derived historical MJO index. We transformed this daily ship observation data into 4° × 4° spatial gridded data. All MJO days having amplitude more than 1.5 times standard deviation for the 1979–2008 period (which is approximately equal to 1.0 for WH04 RMM index) are considered for preparing the cloud fraction composites.

## Supplementary information

Supplementary Information

## Data Availability

The WH04 index from 1974 to 2015 is obtained from the Australian Bureau of Meteorology (https://www.bom.gov.au/climate/mjo/) and the OT12 index from 1905 to 2015 is obtained from Eric Oliver ( https://ecjoliver.weebly.com/mjo-reconstruction.html). The JRA55 MJO index from 1958 to 2015 derived by Klotzbach et al.^13^ is used in the present study. The monthly PDO index from 1905 to 2015 is provided by Washington University (https://research.jisao.washington.edu/pdo/PDO.latest). The PDO index is prepared based on UKMO Historical SST (1900–81)^36^, Reynold's Optimally Interpolated SST (OISST) version 1 (1982–2001)^37^, and version 2 datasets. Sea level pressure (SLP) is obtained from the NOAA-CIRES-DOE Twentieth Century Reanalysis (20CRV3) datasets^38,39^. Here, we use the ensemble mean SLP data derived from the 80 ensemble members of the 20CRV3 reanalysis dataset. OT12 computed the MJO index for each of the 54 ensemble members (SLP) of the 20CRV2 data and prepared an ensemble mean MJO index. However, we computed the MJO index from the ensemble mean SLP data. We have used the ensemble mean SLP data from 1905 to 2015 and reconstructed the MJO index during this period. We have not reconstructed the MJO index before 1905 due to the lack of reliability of the SLP data before 1905. We prepare composites of cloud fraction for each of the MJO phases for the verification of our derived historical MJO index. The cloud fraction data is obtained from Extended Edited Synoptic Cloud Reports (EESCR) dataset, which is based on the ship observations during the 1952–2008 period.

## Code availability

The ML codes and the derived MJO indices (CNN-1D, SVR, MLR, CNN-2D) used in the current analysis are available at https://github.com/abirmetya/MJO_ML.git.
