# Supplementary material for: Exploring the long-term changes in the Madden Julian Oscillation using machine learning
Source: Sci Rep. 2020 Oct 29;10:18567. doi: 10.1038/s41598-020-75508-5 (PMC7596094; doi:10.1038/s41598-020-75508-5)
Supplement: Supplementary file 1 — Supplementary Information [file 41598_2020_75508_MOESM1_ESM.docx]

Supplementary Information

**Exploring the long-term changes in the Madden Julian Oscillation using machine learning**

**Panini Dasgupta**^1,2*^, **Abirlal Metya**^1,3^**, C. V. Naidu**^3^**, Manmeet Singh**^1,4^**, M K Roxy**^1^

^1^ *Centre for Climate Change Research, Indian Institute of Tropical Meteorology, MoES, Pune 411008, India*

^2^ *Department of Meteorology and Oceanography, College of Science & Technology, Andhra University, Visakhapatnam, Andhra Pradesh 530003, India*

^3^ *Department of Atmospheric and Space Sciences, Savitribai Phule Pune University, Pune, Maharashtra 411007, India*

^4^ *IDP in Climate Studies, Indian Institute of Technology Bombay, Mumbai, India*

^*^*Correspondence to panini.dasgupta@tropmet.res.in*

**Supplementary Figure:**

**
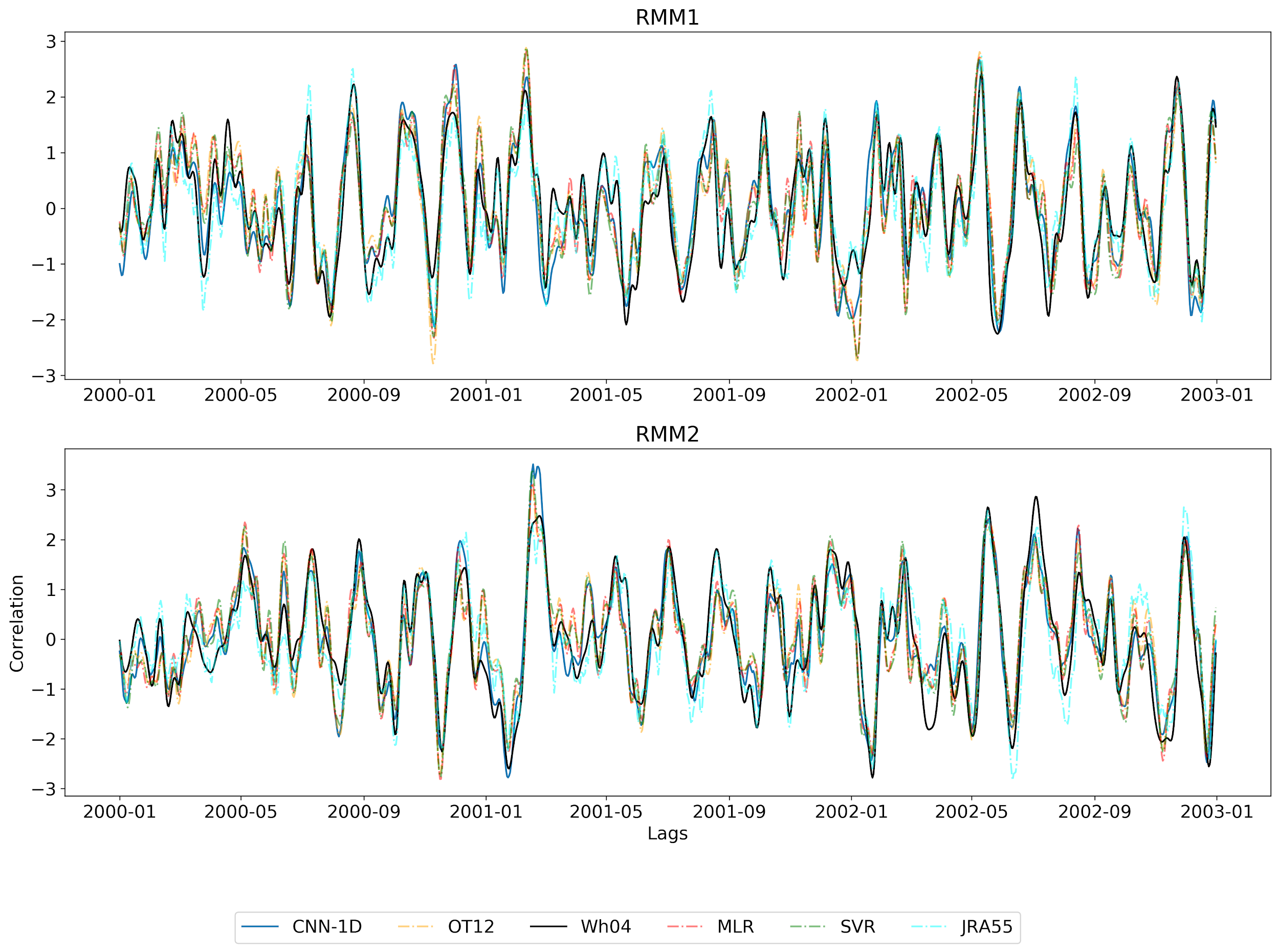
**

**Supplementary Figure 1:** Time series of RMM1 (upper panel) and RMM2 (lower panel) using CNN-1D (blue), OT12 (orange), WH04 (black), MLR (red), SVR (green) and JRA55 (cyan) index.


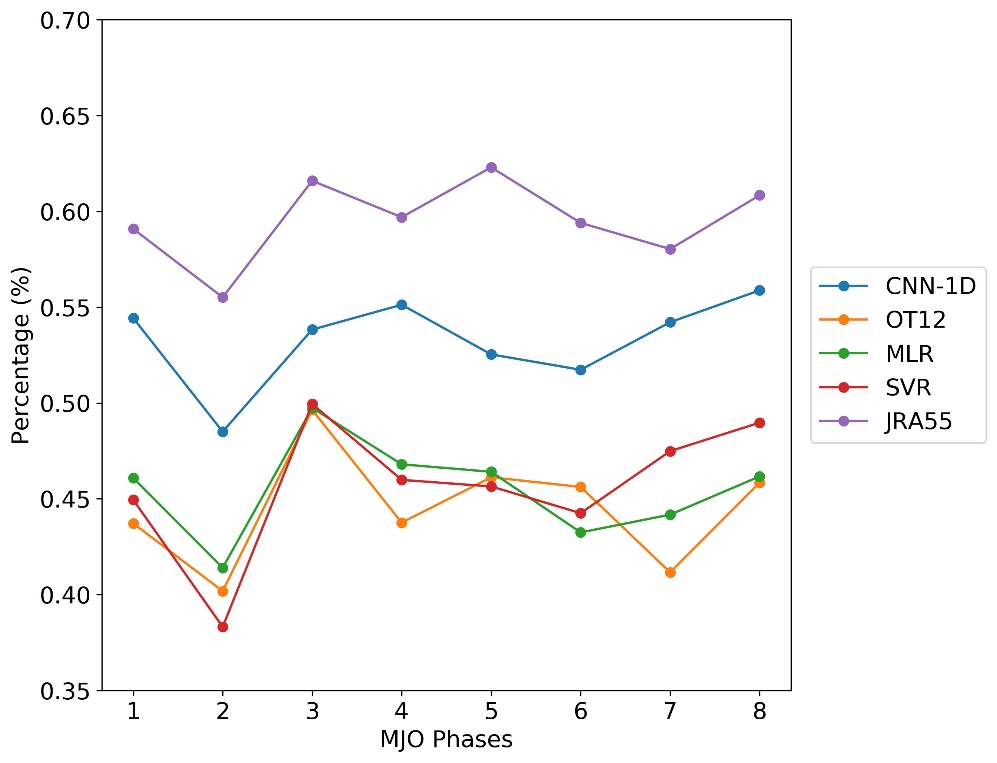


**Supplementary Figure 2:** The accuracy (in %) with which different MJO indices (CNN-1D, OT12, SVR, MLR, JRA55) represent the correct MJO phase at each time step (represented by the original WH04 MJO index) in the known period (1979-2014). This figure explains the fidelity of different MJO indices. Notably, there are substantial improvements in the MJO phase-agreement in the CNN-1D index than the earlier OT12 index.


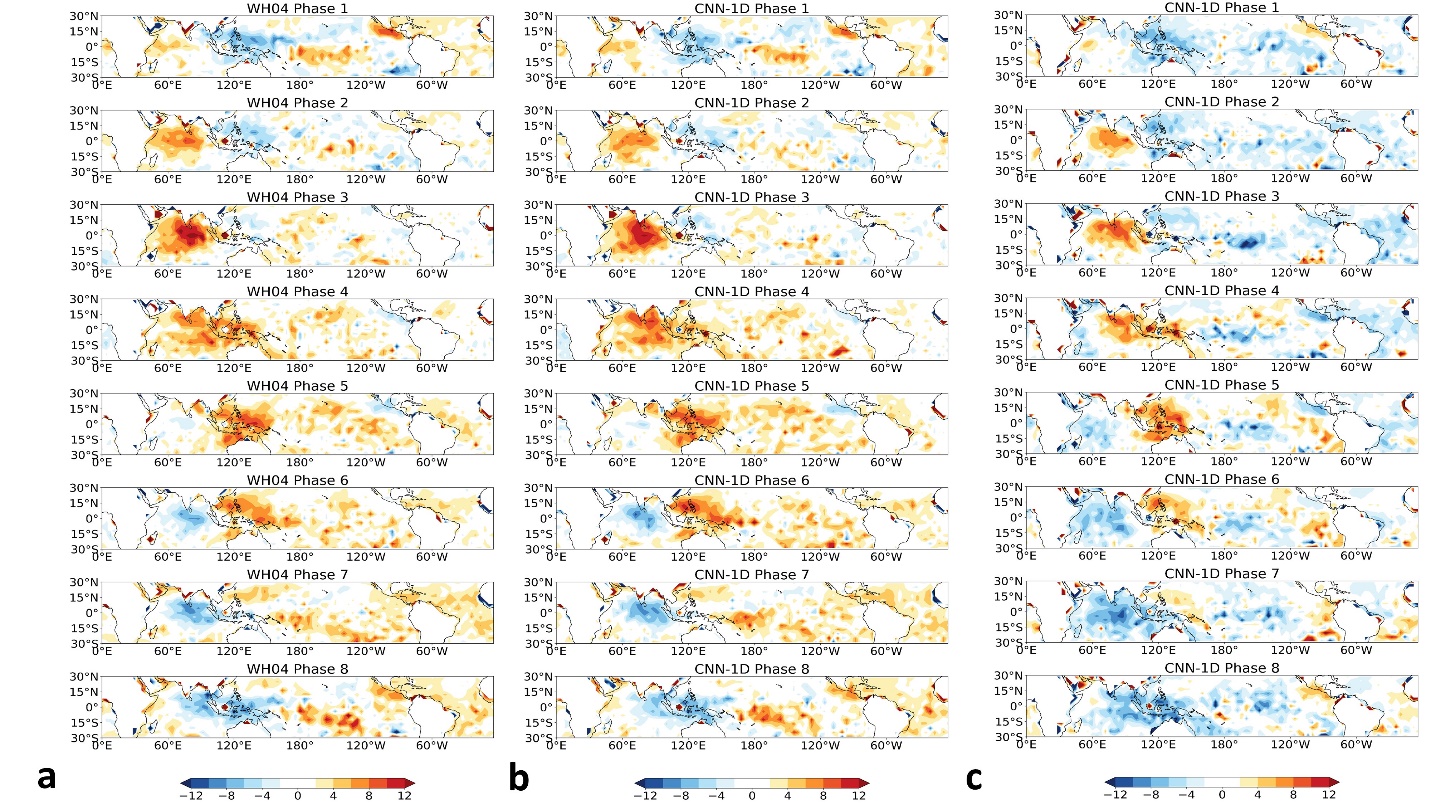


**Supplementary Figure 3:** Cloud fraction composites for different MJO phases. EESCR cloud fraction composites for MJO days when MJO amplitude is greater than 1.5 times of its standard deviation during the period 1979–2008. Composites based on (a) WH04 RMM (b) CNN-1D RMM during the satellite era (1979–2008) and (c) CNN-1D RMM during the pre-satellite (1952–1978) era.


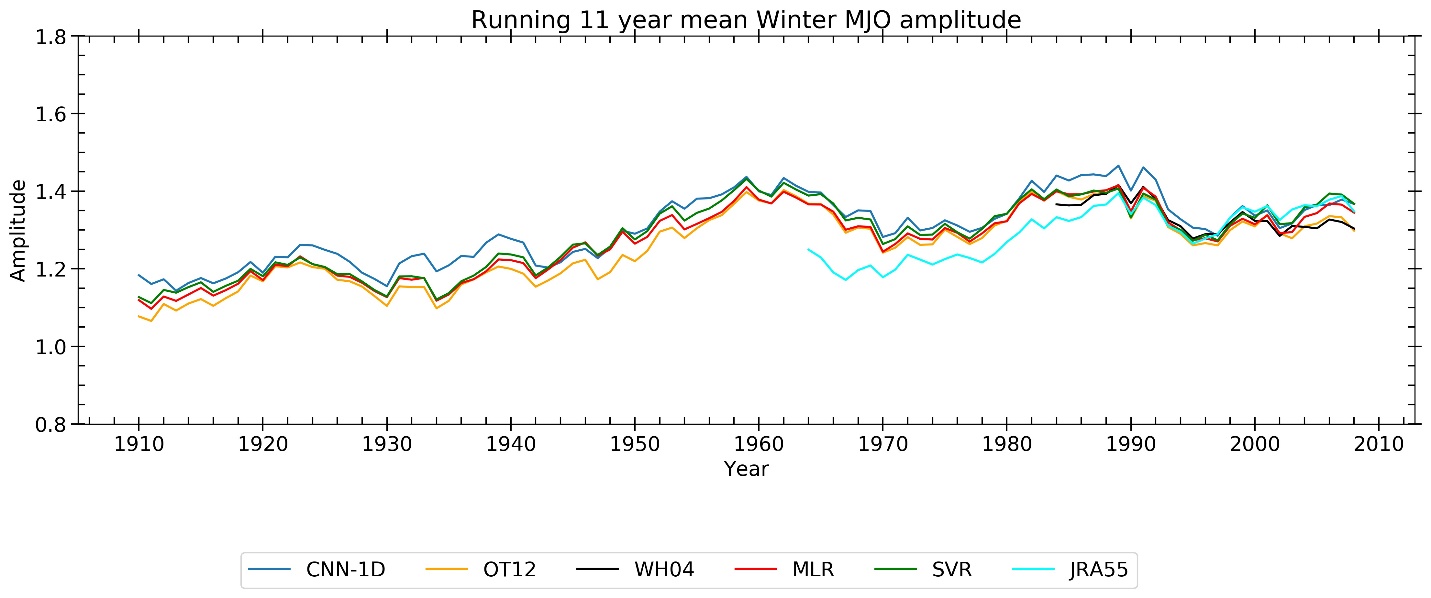


**Supplementary Figure 4:** Running 11-year mean boreal winter MJO amplitude for CNN-1D, OT12, WH04, MLR, SVR, JRA55 MJO index (blue, orange, black, red, green and cyan respectively).

**Supplementary Table:**

**Supplementary Table1**: Locations of 12 predictor points, based on[^1^](https://www.zotero.org/google-docs/?vUk7Ix).

| **Location** | **Longitude (°E)** | **Latitude (°N)** |
| --- | --- | --- |
| **1** | 106 | -6 |
| **2** | 86 | 0 |
| **3** | 326 | -14 |
| **4** | 114 | 14 |
| **5** | 336 | 14 |
| **6** | 56 | 0 |
| **7** | 90 | 14 |
| **8** | 140 | -14 |
| **9** | 318 | 0 |
| **10** | 274 | 6 |
| **11** | 8 | 0 |
| **12** | 130 | 0 |

**References**

[1. Oliver, E. C. J. & Thompson, K. R. A Reconstruction of Madden–Julian Oscillation Variability from 1905 to 2008. *J. Clim.* **25**, 1996–2019 (2012).](https://www.zotero.org/google-docs/?aJULAe)
